# Determinants of differences in RT-PCR testing rates among Southeast Asian countries during the first six months of the COVID-19 pandemic

**DOI:** 10.1371/journal.pgph.0002593

**Published:** 2023-11-07

**Authors:** Michael Van Haute, Alexandra Agagon, Franz Froilan Gumapac, Marie Abigail Anticuando, Dianne Nicole Coronel, Mary Coleen David, Dan Ardie Davocol, Eunice Jairah Din, Carlos Alfonso Grey, Young Hee Lee, Marvin Bryan Muyot, Charissma Leiah Ragasa, Genesis Shao, Cailin Adrienne Tamaña, Trixia Scholastica Uy, Jeriel De Silos

**Affiliations:** College of Medicine, De La Salle Medical and Health Sciences Institute, City of Dasmariñas, Cavite, Philippines; PLOS: Public Library of Science, UNITED STATES

## Abstract

A positive correlation has been demonstrated between gross domestic product (GDP) per capita and COVID-19 tests per 1000 people. Although frequently used as an indicator of economic performance, GDP per capita does not directly reflect income distribution inequalities and imposed health costs. In this longitudinal ecological study, we aimed to determine if, besides GDP per capita, indicators relating to governance, public health measures enforcement, and health and research investment explain differences in RT-PCR testing rates among countries in Southeast Asia (SEA) during the first six months of the COVID-19 pandemic. Using open-access COVID-19 panel data, we estimated the effect of various indicators (GDP per capita, health expenditure per capita, number of researchers per one million population, corruption perceptions index, stringency index, regional authority index) on daily COVID-19 testing by performing fixed-effects negative binomial regression. After accounting for all indicators, the number of daily confirmed COVID-19 cases, and population density, the model provided a 2019 GDP per capita coefficient of 0.0046330 (95% CI: 0.0040171, 0.0052488; *p* <0.001), indicating that a rise in 2019 GDP per capita by 100 international dollars is associated with a 46.33% increase in the number of daily tests performed. Additionally, all indicators were significantly associated with the daily number of RT-PCR testing on multivariable analysis. In conclusion, we identified different country-level indicators significantly associated with differences in COVID-19 testing rates among SEA countries. Due to the study’s ecological design, we caution on applying our results to the individual level given potential for systematic differences between the included countries. Additional investigation is likewise needed to understand how government expenditure on healthcare may have impacted COVID-19 testing capacity during the initial stages of the pandemic.

## Introduction

COVID-19 was first identified as a cluster of pneumonia cases in Wuhan, China in December 2019. The World Health Organization (WHO) officially declared the novel coronavirus outbreak a global pandemic on 11 March 2020. By that time, the causative pathogen, SARS-CoV-2, has already affected 114 countries, infecting over 118,000 people and causing almost 4,300 fatalities in its wake [1]. Following this declaration, many countries began implementing nonpharmaceutical interventions (NPIs, which include lockdown and quarantine measures, school and public venue closures, activation of curfews, banning of social gatherings, and suspension of business activities) at varying degrees. A major part of the WHO’s pandemic response was to accelerate research and development of vaccines, and to launch a worldwide clinical trial initiative intended to find effective treatments [2]. Apart from proper hand hygiene, physical distancing, and travel restrictions, the WHO strongly recommended active case-finding and case management via contact tracing and swab-testing using reverse transcriptase polymerase chain reaction (RT-PCR) [1], all in efforts to suppress and contain COVID-19, thereby reducing its health and socioeconomic consequences.

COVID-19 entry into Southeast Asia (SEA) was confirmed when the first case was detected in Thailand on 13 January 2020. Pre-pandemic, the SEA region’s rising economy (driven by a combination of domestic consumption, foreign investment, and exports) saw an optimistic outlook notwithstanding a then-forecasted slight drop in real gross domestic product (GDP) growth to 6.1% from 6.3% in 2019 [3]. Despite these encouraging figures, there emerged notable differences in COVID-19 testing rates across SEA countries. Media outlets invoked differences in leadership styles, testing approaches (i.e., mass testing versus targeted testing of suspected and probable cases and their contacts), efficiency of RT-PCR kit procurement and validation, speed of laboratory facility accreditation, and timeliness of RT-PCR rollout as possible explanations for these testing disparities. While some nations took the initiative of developing and manufacturing their own testing kits, others remained largely reliant on purchased or donated kits from abroad. Countries that initially had the slowest testing rates began ramping up their testing capacities thereafter, though it took a significant amount of time before they caught up with countries that were already testing their population at a larger scale. Before that, the limited extent of testing grossly underestimated the true number of cases, presumably by a factor of 10 [4].

A positive correlation exists between SARS-CoV-2 RT-PCR tests performed per 1000 people and gross domestic product (GDP) per capita when both axes are set at the logarithmic scale [5, 6]. This relationship inspired the question of whether other country-specific factors could have possibly accounted for these testing disparities as well. At first glance, this relationship entails that countries enjoying greater economic prosperity see a higher rate of testing done, presumably by being able to secure enough testing kits and appropriate laboratory facilities and equipment for their use. While GDP per capita is a frequently used indicator of economic performance and welfare, it has its share of shortcomings. Apart from being a lagging economic indicator (i.e., it reflects the economic performance of a country for a specific period that has already occurred, usually a year), it also does not directly reflect the inequalities in a country’s income distribution, or the costs imposed on human health [7]. Additionally, information on indicators that can quantify this degree of inequality (e.g., the Gini coefficient) are either unreported or not updated for some countries [8]. In middle- to lower-income countries, more patients still tend to pay out-of-pocket for laboratory diagnostics. Similarly, how much a government invests in research and development may be indicative of a country’s readiness in developing its own testing kits. Finally, quality of public services, government credibility, and formulation and effective implementation of policies to address the pandemic also play vital roles in carrying out the much-needed viral testing in a timely manner.

In this ecological study, we aimed to determine if, besides GDP per capita, country-level indicators relating to governance, enforcement of public health measures, and government investment in health and research significantly influenced the observed differences in COVID-19 testing rates among SEA countries during the first six months of the pandemic.

## Methods

### Study design, setting, and data sources

This is a longitudinal ecological study that made use of open-access data obtained from Our World in Data (OWID) [5] (licensed under Creative Commons and stored in repositories provided by GitHub [9], https://github.com/owid/covid-19-data/tree/master/public/data), World Bank Open Data [10] (https://data.worldbank.org), Transparency International (https://www.transparency.org/en), and the Cadmus European University Institute Research Repository [11] (https://cadmus.eui.eu). In accordance with our problem statement and study objective, we limited our scope to data obtained from the eleven SEA countries (Brunei, Cambodia, Indonesia, Laos, Malaysia, Myanmar, Philippines, Singapore, Thailand, Timor-Leste, and Vietnam), and limited the period of our analysis to the first six months of the pandemic, starting on and including 13 January 2020. We concentrated on the first six months of the COVID-19 pandemic because it was during this period when governments of different nations were at the height of mobilizing resources to implement planned public health measures in coordination with the different sectors in the race to control virus spread, whilst periodically evaluating and modifying these implementations to come up with more effective plans of action. SEA has held significant importance during the COVID-19 pandemic due to factors directly and/or indirectly related to pandemic response, hence this study’s focus on the region. First, SEA is strategically positioned at the crossroads of major international trade routes, making it an important hub for global commerce. The region’s proximity to China made it one of the first areas to be affected by the SARS-CoV-2 virus. The high population density of many cities such as Jakarta, Bangkok, Manila, Singapore, and Ho Chi Minh City likewise presented unique challenges in containing virus spread. Second, SEA countries have a history of regional cooperation, exemplified by the Association of Southeast Asian Nations (ASEAN), which played a critical role in managing the pandemic by facilitating information sharing, coordinating responses, and implementing regional initiatives to combat COVID-19 [12, 13]. Third, the region’s prior experiences with pandemics, such as SARS in 2003 and H1N1 in 2009, enabled them to develop essential systems for preparing and responding to pandemics. These mechanisms were swiftly put into action by health authorities as soon as they received news about the novel coronavirus originating in China. Consequently, the region achieved relatively better results in controlling viral transmission and maintaining low fatality rates, albeit at the expense of economic growth [12]. Lastly, SEA countries are geographically diverse, with Laos being landlocked, Indonesia, the Philippines and parts of Malaysia existing as archipelagos, and the rest having contiguous coastlines and bounded by other SEA countries at the same time. Apart from this geographical diversity, several SEA countries have varying degrees of government decentralization [14]. Both factors play a role in the extent of border control particularly during outbreaks.

### Dependent variable

The dataset obtained from OWID includes panel count data on cumulative and daily number of confirmed COVID-19 cases, COVID-19-related deaths and RT-PCR tests performed, as reported by the respective health departments of each affected country (a list of country-specific data sources is available from https://ourworldindata.org/covid-testing#source-information-country-by-country). Of these parameters, we used panel count data on *daily RT-PCR tests performed* to represent testing rate.

### Explanatory variables

Information on country-level indicators was collected from World Bank Open Data [10], except for corruption perceptions index (obtained from Transparency International), stringency index (obtained from OWID), and regional authority index (obtained from the Cadmus European University Institute Research Repository).

#### GDP per capita

GDP per capita is an economic indicator that breaks down a country’s GDP (defined as the total value of goods and services produced within one year [15]) per person by dividing it by its population, and is expressed in international dollars, a hypothetical currency that, when used in a particular country, would buy a comparable amount of goods and services one US dollar would buy in the United States after adjusting for purchasing power parity (PPP) and international average prices of commodities. In the context of the SARS-CoV-2 pandemic, this indicator can arguably be used as a proxy for a country’s ability to procure test kits and mobilize human resources, suppliers, and equipment to do RT-PCR testing, while also ensuring that the health system can still provide good quality clinical and public health interventions for non-COVID-19 cases.

As earlier mentioned, GDP per capita is typically considered a lagging economic indicator wherein healthcare system financing for a particular year would depend on the mobilization of resources made during the preceding year. For that matter, *GDP per capita of 2019* (instead of 2020) was used to represent the main explanatory variable in this study. As economic trends from the beginning of 2020 onward could still influence the daily number of RT-PCR tests performed, albeit to a limited extent if any, the association between 2019 GDP per capita would be best interpreted while controlling for *GDP per capita of 2020* in the analysis.

#### Indicators on government health and research investment

Health expenditure (HE) reflects the final consumption of healthcare goods and services, with these goods pertaining to either commodities necessary for personal health or collective services provided by the government to its citizens [16]. The percentage of GDP spent on healthcare is set in the budget of each government for the year. The budget for the Ministry/Department of Health of each country is meant to fund the various healthcare programs and services that it normally provides the population. In the SEA region, both public and private sectors fund healthcare spending, with the former in the form of government spending and compulsory health insurance contributions, and the latter as contributions from private health insurances, non-governmental organizations, and other private corporations, together with out-of-pocket expenses [17]. While health systems of SEA countries have a dynamic mix of both public and private sectors participating in delivery and financing healthcare services [17], there has been a general shift towards the private sector dominating healthcare financing [18]. The push towards universal health coverage (UHC) in the region is likewise in progress, but the pace is varied and uneven that the poor and/or uninsured still find themselves paying mostly out-of-pocket [18, 19]. Even in the pre-pandemic era, healthcare systems of some poorer countries have already been struggling to provide public health services due to fragmented healthcare financing, with out-of-pocket spending representing at least 60% of total healthcare expenditure on average [20]. As mentioned, majority of SEA countries were forecasted to experience a drop in GDP per capita during the initial period of the pandemic. In this regard, patients needing to pay out-of-pocket for their healthcare generally become reluctant to seek medical help due to high cost of testing alone if they start manifesting symptoms of COVID-19, thereby hampering timely disease detection and documentation. Moreover, difficulties in engaging the private sector to provide appropriate health statistics, which can eventually lead to disease underreporting, undermine disease surveillance networks particularly during disease outbreaks [21, 22]. Similarly, inadequate government healthcare spending would invariably mean straining already limited resources, particularly for countries that spend 5% or less of their gross national product on healthcare whilst striving towards UHC [23]. The COVID-19 pandemic is an unexpected health crisis and is therefore beyond the capacity of the usual or normal budget to address. Nonetheless, robust economies (as reflected by strong GDPs per capita) may have better capacities to cope with the crisis in that they may be able to come up with supplemental (i.e., emergency or contingency) budgets to finance the requirements needed to better face the health problem. Moreover, the availability of supplemental emergency funds is a fundamental significant component of government pandemic preparedness, if any is in place. Additionally, countries with robust GDP growth tend to have better credit ratings and therefore may be able to secure loans to fund programs and services to meet health emergencies. In the context of this current pandemic, this would cover the direct costs of performing SARS-CoV-2 RT-PCR tests besides the costs of medications, healthcare personnel staffing and use of tools such as mechanical ventilators and personal protective equipment. Indirect or overhead costs allocated for securing testing kits, along with constructing, accrediting, and maintaining COVID-19 laboratory testing facilities, likewise add to spending on services intended for collective consumption. For this study, we posited that the higher the HE allocated, the higher the RT-PCR testing rate. The most likely pathway for this relationship is adequate HE enabling governments and health systems to allocate emergency funds needed to invest in the necessary infrastructure for testing (i.e., setting up testing centers, acquiring testing kits and supplies, establishing laboratory facilities, and ensuring the availability of trained personnel to conduct tests), ensuring that RT-PCR testing is accessible to a large portion of the population (via subsidization making it affordable for individuals who may otherwise hesitate to get tested due to financial constraints), and enabling testing capacity scale-ups by investing in additional testing equipment, hiring and training more healthcare professionals, expanding laboratory facilities, and deploying mobile testing units to reach underserved areas or populations. In our analysis, we used the indicator *HE per capita*, likewise expressed in international dollars adjusted for PPP.

Closely related to health spending is investing money and manpower on research and development (R&D). Tenably, higher levels of R&D expenditure make for a sound productive system of innovation [24]. In the context of the SARS-CoV-2 pandemic, this would translate to timely development and rollout of RT-PCR testing kits, and thus would make sense to include R&D expenditure in the analysis. At the onset, however, there already existed a large disparity in laboratory capacities among the SEA countries, with Singapore having the most advanced research capacity, and Myanmar, Laos, and Cambodia still struggling with providing basic laboratory services in related fields such as HIV/AIDS and hepatitis [25]. Several large institutions in Malaysia, Thailand, and Indonesia are equipped with sophisticated molecular biology tests, and these may not necessarily have been funded by their respective governments. In this regard, R&D expenditure may not necessarily reflect a nation’s capacity to cope with the pandemic as each may have different research agendas to focus on, such as energy development and military buildup. Moreover, R&D public budget for many countries generally requires a minimum of one year for execution, which would mean that any figure indicated for the year 2020 may not reflect the actual amount of R&D investment, particularly during the early period of the SARS-CoV-2 pandemic. On a similar note, a high number of active researchers working on new knowledge, technologies, and solutions to manage COVID-19 cases is expected to lead to improvement in the number of recoveries and reduction of deaths, provided a working and practical solution was arrived at. Assuming there are epidemiologic or data science-related research being conducted in a particular country, this indicator may also affect how data on COVID-19 is being processed. More researchers working on such data may lead to better data cleaning and processing, thereby reducing the delay in data validation and reporting. The WHO, recognizing the importance of R&D being integral to strategies combating COVID-19, activated the R&D Blueprint [26], a pandemic response initiative intended to accelerate the development of COVID-19 diagnostics, vaccines, and therapeutics through funding of priority research. Thus, we posited that a greater *number of researchers per one million population* contributes to increasing RT-PCR testing rates through the availability of a larger pool of scientific expertise in the understanding of the novel coronavirus’ pathogenicity and in the development of methods that duly detect relevant SARS-CoV-2 viral particles. With better comprehension of the disease mechanism and fostering of collaboration and knowledge-sharing between countries, test development and validation, as well as standardization and optimization of RT-PCR testing protocols is facilitated. Additionally, a higher magnitude of expertise helps streamline the testing process to increase efficiency, ensuring countries to rapidly expand their testing capacity by allowing test developers to train laboratory personnel, provide technical guidance, and facilitate the implementation of standardized protocols.

#### Indicators on governance and public health measures enforcement

The *corruption perceptions index* (CPI), formulated and published annually by the non-governmental organization Transparency International [27], is a standardized metric ranging from 0 (“highly corrupt”) to 100 (“very clean”) that ranks different countries according to how corrupt their public and business sectors are perceived to be based on several opinion surveys and expert assessments on corruption collected from institutions such as the World Bank, the World Economic Forum, and various other think tanks. It captures various facets, including bribery, diversion of public funds, integrity of government officials and government sectors, capability of the government to hide corruption, red tape, nepotism, prosecution of corruption cases, protection given to media reporting cases of corruption, and transparency of public interest information [28]. Observation of a strong positive rank correlation between GDP per capita (considered an unambiguous result of objective quantitative measures) and CPI suggest that the latter’s values are not based on subjective evaluation [29, 30]. Additionally, less corrupt countries appear to exhibit significant long-term economic growth [29], with an average increase in annual GDP per capita growth rate by 1.7% occurring for every unit increase in CPI [31]. While the concept of less corrupt countries faring better comparatively in terms of testing, recording, and reporting COVID-19 data is an attractive initial supposition, how CPI and RT-PCR testing rates are related may be more complex and multifaceted than anticipated that this relationship should be tackled in light of principles such as accountability, transparency, public trust, stringency of government policies, and the extent of authority devolution to subnational or regional governments (discussed next).

It is widely recognized that in any health crisis, such as the SARS-CoV-2 pandemic, the government is expected to play a crucial role in providing healthcare services, economic assistance, coordination of relief efforts, and other forms of public support without misappropriation of public funds. The importance of government support in overcoming health crises effectively and rapidly aligns with the general understanding of disaster management. Governments are called upon to respond effectively and inclusively, and are obligated to make decisions based on scientific data rather than political opportunity [32]. A major part of this decision-making process involves timely procurement and rollout of RT-PCR testing kits. However, during the COVID-19 pandemic, many of these decisions ended up focusing largely on political and economic considerations [32], with imposition of NPIs and changes in economic practices becoming conducive media for corruption to worsen [33, 34]. Any complicity that resulted from corruption led to increases in COVID-19 public health risk exposure and confirmed cases [35, 36] that further burdened already weakened healthcare delivery systems. Ineffective pandemic responses naturally eroded much of public trust, which fostered suspicion towards political institutions and non-compliance with imposed pandemic policies accordingly [37]. Thus, accountability and transparency become essential to ensure public trust [38, 39]. By conveying the government’s actions and decision-making processes transparently, the public can better understand the steps taken in tackling the pandemic. This transparency allows for accountability (where the government can be held responsible for its actions or lack thereof), as well as enables the public to participate in the decision-making process and provide valuable input [32]. Additionally, the degree of political accountability increases when punishment of corrupt individuals is encouraged, and if informational problems relating to government activities are reduced [40]. Evidence demonstrates that promoting accountability and transparency practices is crucial for maintaining public confidence and facilitating a more coordinated response during health crises [41]. Transparency likewise extends to providing adequate information to the public regarding RT-PCR test availability and the government’s capacity to handle demands for testing. During the early stages of the pandemic, accountability also became essential in ensuring that testing facilities were adequately prepared, well-equipped, and capable of providing accurate and timely results. Restoring and maintaining public trust through transparent and accountable actions of authorities became crucial to ensure widespread acceptance and participation in RT-PCR testing initiatives.

Conceivably, countries with higher levels of corruption may have weaker healthcare systems, including less investment in medical infrastructure, equipment, and personnel, which could impact their ability to develop and maintain adequate testing capacity for COVID-19. Additionally, corrupt practices such as embezzlement, bribery, and kickbacks may divert resources away from public health efforts, which include timely RT-PCR testing. On the other hand, officials of countries with high levels of corruption would allocate more resources to testing and other public health efforts as a means of maintaining their power and legitimacy. In these cases, corruption may not necessarily lead to lower testing capacity. From a different perspective, citizens would trust its government policies more if corruption is less. Morris and Klesner [42] describe a powerful mutual causality between corruption perceptions and public trust in political institutions. In the context of the COVID-19 pandemic, greater public compliance to NPIs and public health measures was observed when government trust was substantial [43]. This dovetails with high public trust amplifying compliance with imposed COVID-19 health guidelines even with increasing levels of stringency [44]. When the pandemic began, most governments mandated large-scale policies that included several population mobility-restricting NPIs. Stricter intervention measures implemented during the early months of the pandemic were associated with reduced viral transmission [45] and lower rates of COVID-19-related deaths [46]. While national governments laid down these measures, the task of implementing them generally got delegated to subnational government units in many countries. Local governments took several epidemiological and socioeconomic factors into consideration to maximize the chances of effective implementation of these mandates, as they were often left to deal with limited resources, particularly in rural or geographically isolated communities where access to proper health facilities is still inadequate, thereby inadvertently making them the direct bearers of the pandemic’s economic, social, and fiscal brunt [47]. In less developed and deprived enclaves where pandemic preparedness tended to be poor, COVID-19 policy implementation was reluctantly laxer, as severely limiting their population’s mobility would mean crippling their local economy [48]. While such setup is anticipated to create a compromising scenario wherein devolution of authority to subnational governments becomes fertile ground for corruption, theoretical and empirical studies investigating the relationship between decentralization and corruption yielded mixed results [49]. However, these studies were criticized for predominantly concentrating on examining the impact of fiscal decentralization on corruption while largely overlooking organizational structures and dynamics of national parties. A more recent study [50] looked into how political context factors into corruption and found that the presence of local elections and party institutions decentralized from national control compel locally elected officials to be more accountable and transparent toward their constituents, which could possibly reduce levels of corruption.

While concurrently controlling for the independent effects of transparency, accountability and public trust on the outcome is desired to be able to describe a more informative relationship between corruption and RT-PCR testing rates, corresponding established country-level indicators are either unavailable altogether, or have missing values for some countries (e.g., T-index [51], open government index [52]). On the other hand, the *stringency index* (SI), formulated by the Oxford Coronavirus Government Response Tracker (OXCGRT) Project [53], is a widely available composite measure that equals the average score from nine response metrics (namely: school closures; workplace closures; cancellation of public events; restrictions on public gatherings; closures of public transport; stay-at-home requirements; public information campaigns; restrictions on internal movements; and international travel controls), each ranging from 0 to 100, with a higher score indicating a stricter response. While the authors of the SI warn that this indicator merely records the number and strictness of government policies and does not quantify appropriateness or effectiveness of governments’ COVID-19 pandemic responses, higher stringency was nonetheless related to lower daily new COVID-19 infections [54, 55] and lower COVID-19-related death rates [40, 55]. With lesser degrees of exposure and infections resulting from scenarios where public trust and compliance are high, one can posit that lesser amounts of testing are probably needed in such scenarios. Another composite measure, *regional authority index* (RAI), attempts to capture the extent of government decentralization by quantifying the authority exercised by a country’s subnational or regional governments (if any) in terms of “self-rule” (authority that a subnational/regional government can exercise within its own jurisdiction based on its institutional depth, policy scope, fiscal authority, borrowing authority, and extent to which it has autonomous, elected representation) and “shared rule” (authority that a subnational/regional government can co-exercise and co-determine national legislation, executive policy-making, tax allocation, borrowing constraints, and constitutional reform) [14]. This metric’s value can range from 0 to 30, increasing in proportion to the degree of decentralization. While the RAI itself may also run a similar risk of oversimplifying the complex nature of decentralization in its attempt to capture the political, fiscal, and administrative aspects of delegated authority into a single index, especially for authoritarian regimes, the disaggregated approach to determining this metric allows for presenting abstract concepts into qualities that can be empirically assessed against a particular country’s history as reflected by its constitution, laws, executive orders, and political context [14]. With that, a higher degree of authority devolution to subnational governments, which could translate to greater accountability afforded to locally elected officials, may also turn out to facilitate distribution of goods and services (including SARS-CoV-2 RT-PCR tests) to provinces or rural areas rather than being concentrated in the metropolis. Overall, by accounting for the independent effects of stringency and decentralization on RT-PCR testing rates through inclusion of SI and RAI as covariates in our analysis, we may arrive at a more accurate relationship between the degree of perceived corruption and RT-PCR testing rates.

### Other variables

The *number of daily confirmed COVID-19 cases* (obtained from OWID) was included as another covariate in our analysis. Information on the trend of daily new cases provided by this measure may reflect pandemic severity and may have a more direct bearing on the frequency of COVID-19 testing. When there is an upward trend in the number of new COVID-19 cases, a corresponding increase in demand for RT-PCR testing is generally expected. In countries that performed strongly in terms of pandemic response, disease containment was better when high levels of testing were carried out early in the health crisis, with testing capacities increased accordingly in response to increasing number of cases [56]. Depending on how much additional resources public health authorities and policymakers are able to allocate, a higher COVID-19 caseload can lead to increased testing capacity that may not necessarily be accompanied by implementation of targeted testing strategies. On the other hand, an increasing case trend can also put a strain on testing resources, including staffing, equipment, and supplies, leading to delays in testing, long wait times for results, and eventual reduced capacity for testing. An important caveat to using daily new COVID-19 cases in the analysis is that pandemic severity can likewise vary by geographic area, as different areas may have different levels of healthcare capacity, demographics, and public health policies.

Another variable of interest is *population density*. For high-population density countries, where higher SARS-CoV-2 transmission and COVID-19-related mortality were frequently observed [57, 58], mass testing to achieve prompt case identification, quarantine, and eventual transmission chain interruption has been the benchmark recommendation to avoid economic and societal disruptions stemming from stringent NPI enforcement. In general, areas with high population density often require more resources to meet the testing needs of their residents, and ideally, it may be necessary to set up multiple testing centers or mobile testing units to ensure that testing is available to everyone. However, for many of these countries, tests had to be performed sparingly during the earlier months of the pandemic because of their limited availability, inevitably making targeted testing their only feasible option [59]. All these considered, modeling the number of daily RT-PCR tests performed taking population density into account would hope to capture this on-the-ground experience to come up with more informative estimates of its relationship with GDP per capita. Since population density can be used as a measure of spatial distribution of inhabitants within an area, it may tenably represent an area of space in which new COVID-19 cases are generated. For that matter, population density was identified as the exposure variable in our analysis (wherein the software will include the natural logarithm of this variable in the fixed-effects model with coefficient constrained to 1). Information on country-specific population densities was obtained from World Bank Open Data.

### Data gathering, preparation, and cleaning

Daily and cumulative count data on RT-PCR testing and COVID-19 confirmed cases, along with their respective dates, were first extracted from the full dataset downloaded from OWID. These were then grouped by country, tabulated chronologically on separate spreadsheets, counterchecked against the data reported by the listed country-specific data sources (whenever available), and assessed for completeness. Following this initial data processing, the different spreadsheets were combined into one dataset. Afterwards, country-specific data on population density, GDP per capita, HE per capita, number of researchers per one million people, CPI, SI, and RAI were added to comprise the final dataset for analysis.

Missing data on country-level indicators not requiring multiple metrics or complex methods for derivation (i.e., HE per capita, number of researchers per one million people), if any, were imputed by regressing the indicator in question on the other country-level indicators, including population density. Robust regression (*rreg* Stata command) was used for this purpose in anticipation of extreme indicator values, as it is less sensitive to outliers compared to standard linear regression.

### Statistical analysis

All statistical analyses were carried out using Stata 17 (StataCorp, College Station, TX). The total number of RT-PCR tests performed by the end of the six-month period of research interest and GDP per capita were logarithmically transformed, and the correlation between them was determined using Pearson’s correlation method. To estimate the relationship between daily RT-PCR tests and the covariates (GDP per capita, HE per capita, number of researchers per one million people, CPI, SI, RAI, number of daily confirmed COVID-19 cases) while setting population density as the exposure, Poisson regression analysis was performed (*xtpoisson* command, declaring country as the panel variable and days as the time variable). The fixed-effects approach in panel data analysis was utilized to control for possible unmeasured time-varying confounders as well as unobserved time-invariant heterogeneity across countries, thereby reducing omitted-variable bias. Omitted-variable bias occurs when there are unobserved factors that are correlated with both the independent variables of interest and the dependent variable, leading to inconsistent estimates. In Poisson regression, the natural logarithm of the expected count (or log count) of the response variable (i.e., the number of daily RT-PCR tests performed) is modeled as a function of the explanatory variables or covariates. The regression coefficient estimated by the model is interpreted as follows: for every unit change in the explanatory variable, the difference in the expected log count of the response variable is expected to change by the value of the respective regression coefficient, holding all other explanatory variables or covariates constant. For example, if the regression coefficient is 0.015, this means that the difference in the log count would be expected to increase by 0.015 units for every unit increase in the explanatory variable, while holding the other covariates in the model constant. Alternatively, this can be expressed as: for every unit increase in the explanatory variable, the expected count increases by (*e*^0.015^–1) × 100% or ~1.5%. The Poisson model likewise assumes that the distribution of the response variable has a mean equal to its variance, however this assumption is often violated. For this reason, the fitted Poisson model was assessed for overdispersion (i.e., when the variance of the response variable exceeds what is assumed by the model). If overdispersion was present, a fixed-effects negative binomial regression model (*xtnbreg* command) was fitted instead. As with Poisson regression, negative binomial regression also models the expected log count of RT-PCR tests performed as a function of the explanatory variables or covariates. This makes the interpretation of negative binomial regression the same as that of Poisson regression.

Univariable and multivariable analyses were performed. All covariates with statistically significant regression coefficient estimates on univariate analysis were included in the full multivariable regression model. Due to the highly unlikely probability of having any of the explanatory variables equal zero, the intercept term was suppressed in model construction (*noconst* option). Thus, the full model for the daily number of RT-PCR tests performed is specified as follows (where *i* represents country and *t* time): ln(μ_*it*_) = β_1_*[2019 GDP per capita]_*it*_ + β_2_*[2020 GDP per capita]_*it*_ + β_3_*[HE per capita]_*it*_ + β_4_*[Researchers per one million population]_*it*_ + β_5_*[CPI]_*it*_ + β_6_*[SI]_*it*_ + β_7_*[RAI]_*it*_ + β_8_*[Daily confirmed COVID-19 cases]_*it*_ + 1*ln([Population density]_*it*_) + α_*i*_ + u_*it*_. The ln(μ_*it*_) term represents the natural logarithm of the expected count/number of daily RT-PCR tests performed for a given country *i* at time *t* during the study’s 6-month observation period of interest, while the α_*i*_ term captures the unobserved heterogeneity between countries, and u_*it*_ is the idiosyncratic error, also unobserved. Of note, only SI and daily confirmed COVID-19 cases are time-varying in the model, so the number of RT-PCR tests performed during a particular point *t* during the study’s 6-month observation period would depend on the SI and number of confirmed COVID-19 cases during that same time point *t*. For all model-fitting commands, the software employed an iterative technique to locate the maximum likelihood for estimating the parameters of each model constructed. All regression estimates were reported as coefficients along with their corresponding 95% confidence intervals (CI) and *p*-values. Results were considered statistically significant if *p* <0.05.

### Ethics

The study protocol was submitted to the Ethics Review Committee of the De La Salle Medical and Health Sciences Institute–College of Medicine (reference number: CMERC 2021-CM-016) for evaluation. Since the study plan involves the use of open-access secondary country-level indicator data instead of inclusion of human participants, no ethical issue was found by the committee in their assessment, and thus the study was exempted from full ethics review. Because derivation of country-level indicators does not rely on inherent human characteristics, any means by which identities of individual participants during and/or after data collection could be determined is not possible. Thus, obtaining formal informed consent was not necessary.

## Results

Table 1 shows the country-specific total number of RT-PCR tests performed and total number of confirmed COVID-19 cases by the end of the sixth month of the pandemic (dated on 21 July 2020) along with the corresponding population densities and country-level indicators used in the analysis. Given the dynamic nature of the SI in the short term, this indicator was reported as median and range. Cumulatively, 5,679,683 RT-PCR tests were performed among the 11 SEA countries. Indonesia had the highest cumulative total number of RT-PCR tests performed (n = 1,235,545) while Timor-Leste had the lowest (n = 1,290). Setting the total RT-PCR tests performed as of 21 July 2020 and 2019 GDP per capita to the logarithmic scale, the Pearson’s correlation coefficient obtained between them is positive (*r* = 0.4121) albeit not statistically significant (i.e., *r* is not significantly different from 0) with *p*-value of 0.2079. Of note, Timor-Leste did not have information on number of researchers per one million population and was thus imputed by regressing the missing indicator on the other country-level indicators, including population density, as described previously.

**Table 1:** Pertinent country-specific data for each included Southeast Asian country.

Country	Date of first confirmed COVID-19 case	Total RT-PCR tests performed^a^	Total confirmed COVID-19 cases^a^	Population density^b^	GDP, in millions (2019)^c^	GDP per capita (2019)^c^	GDP, in millions (2020)^c^	GDP per capita (2020)^c^	HE per capita^c^^,^^d^	Researchers per one million population^e^	CPI	SI^g^	RAI^h^
Brunei	9 March 2020	34,797	141	84	13,469.24	30,748.3	12,005.83	27,179.4	671.56	284	60	52.78 (19.44–58.33)	0.00
Cambodia	27 January 2020	52,502	197	93	27,089.39	1,671.4	25872.80	1,577.9	113.31	30	21	40.74 (0–68.52)	5.00
Indonesia	2 March 2020	1,235,545	89,869	145	1,119,099.87	4,151.2	1,058,688.94	3,894.3	120.12	396	37	68.06 (28.7–80.09)	20.78
Laos	24 March 2020	23,171	19	32	18,740.56	2,598.5	18981.80	2,593.4	68.22	16	29	56.48 (20.37–96.3)	1.00
Malaysia	25 January 2020	1,053,584	8,815	101	365,177.74	11,132.1	337,337.93	10,160.8	436.61	2,185	51	57.41 (11.11–78.7)	21.48
Myanmar	23 March 2020	104,874	341	82	68,697.76	1,295.2	78,930.26	1,477.5	60.02	32	28	80.56 (54.63–86.11)	12.89
Philippines	30 January 2020	1,148,186	70,764	376	376,823.40	3,413.8	361,751.12	3,224.4	142.08	174	34	83.33 (11.11–100)	11.18
Singapore	23 January 2020	1,009,532	48,434	7,919	376,837.49	66,070.5	345,295.93	60,729.5	2,632.71	7,287	85	50.93 (25–82.41)	0.00
Thailand	13 January 2020	741,202	3,255	140	543,976.70	7,628.6	499,681.76	6,990.9	296.17	1,790	36	36.67 (0–46.53)	4.01
Timor-Leste	21 March 2020	1,290	24	87	2,028.55	1,584.3	2,158.39	1,660.3	92.67	–^f^	40	33.33 (13.89–77.78)	0.00
Vietnam	22 January 2020	275,000	401	308	334,365.26	3,491.1	346,615.75	3,586.3	180.72	757	36	58.33 (0–96.3)	8.00

CPI: corruption perceptions index; GDP: gross domestic product; HE: health expenditure; RT-PCR: reverse transcription polymerase chain reaction; SI: stringency index; RAI: regional authority index

^a^As of 21 July 2020.

^b^Population density is expressed as number of people per square kilometer land area.

^c^Data on GDP, GDP per-capita and HE per capita expressed in terms of international dollars corrected for purchasing power parity.

^d^Latest available World Bank data on HE per capita is as of 2019.

^e^Latest available World Bank data on number of researchers per one million population: Laos = 2002; Brunei = 2004; Cambodia = 2015; Malaysia and Philippines = 2018; Singapore, Thailand and Vietnam = 2019; Indonesia and Myanmar = 2020. No data available for Timor-Leste.

^f^Missing data on number of researchers per one million population for Timor-Leste was imputed by regressing the missing indicator on the other country-level indicators, including population density.

^g^SI data presented as median and range; range contained in parenthesis.

^h^Latest available RAI data from the Cadmus European University Institute Research Repository is as of 2018.

The α dispersion parameter of the fitted Poisson model was noted to be significantly greater than 0 (α = 0.462, 95% CI: 0.211, 1.008; LR test χ^2^ = 3.6 × 10^5^, *p* <0.001), indicating overdispersion. For that reason, a negative binomial regression model was fitted, the results of which are summarized in Table 2. In the univariable analysis, a consistent inverse relationship is observed between each of the indicators and the number of daily tests performed, as evidenced by the negative-signed regression coefficients. The regression coefficient estimates of all covariates were statistically significant on univariable analysis and were thus included in the multivariable analysis. On multivariable analysis, 2019 GDP per capita is positively associated with the number of daily tests performed. Additionally, the inverse relationships observed on univariate analysis were reversed for all other indicators except for 2020 GDP per capita, HE per capita and CPI. Interpreting the regression coefficient for 2019 GDP per capita in the multivariable model (*b* = 0.0046330, 95% CI: 0.0040171, 0.0052488; *p* <0.001), we observe that a rise of 100 GDP per capita international dollars is associated with an expected increase in the number of daily RT-PCR tests by approximately 46.33%.

**Table 2:** Summary of the negative binomial regression analysis showing the association of covariates with number of daily RT-PCR tests performed.

Country-level indicator	Univariable analysis			Multivariable analysis^a^		
	Coefficient, *b*	95% CI	*p*-value	Coefficient, *b*	95% CI	*p*-value
2019 GDP per capita	–0.0000573	–0.0000624, –0.0000521	<0.001	0.0046330	0.0040171, 0.0052488	<0.001
2020 GDP per capita	–0.0000640	–0.0000698, –0.0000582	<0.001	–0.0041574	–0.0049037, –0.0034112	<0.001
Health expenditure (HE) per capita	–0.0017778	–0.0019550, –0.0016005	<0.001	–0.0290574	–0.0315493, –0.0265655	<0.001
Researchers per one million population	–0.0005487	–0.0005950, –0.0005024	<0.001	0.0042798	0.0040215, 0.0045382	<0.001
Corruption perceptions index (CPI)	–0.0270647	–0.0287345, –0.0253949	<0.001	–0.2909428	–0.3011735, –0.2807121	<0.001
Stringency index (SI)	–0.0175658	–0.0187604, –0.0163713	<0.001	0.0380892	0.0349754, 0.0412031	<0.001
Regional authority index (RAI)	–0.0458708	–0.0525743, –0.0391674	<0.001	0.1078193	0.0937510, 0.1218875	<0.001
Daily confirmed COVID-19 cases	–0.0021156	–0.0023650, –0.0018662	<0.001	0.0009416	0.0008342, 0.0010490	<0.001

GDP: gross domestic product; 95% CI: 95% confidence interval

^a^Log likelihood = –8650.6789; Wald χ^2^_df = 8_ = 18982.51, *p* <0.0001

## Discussion

In this ecological study, we attempted to assess if, besides GDP per capita, there were other country-level determinants that could explain the notable differences in RT-PCR testing rates among SEA countries during the first six months of the pandemic. While the positive correlation between the number of RT-PCR tests and 2019 GDP per capita was consistent with published data [5, 6], our computed correlation coefficient, *r*, was not significantly different from 0. This can be explained by the smaller number of countries (n = 11) included in the analysis (as smaller numbers of units of observation generally result in wide confidence intervals that often include the null value). On univariable analysis, 2019 GDP per capita had a negative association with the number of RT-PCR tests. We point out that the modeling methods employed in this study involve panel data, which can capture within-country trends in the daily number of tests performed over time and thus provides an advantage over cross-sectional data. That said, any intermittent rise or fall in testing rates occurring during the period of observation can also be attributable to dynamic factors such as changes in number of daily new infections and shifts in pandemic restrictions imposed in response to surges in infection rates, and these may well be the effects that are picked up and manifested as the negative association between 2019 GDP per capita and testing capacity on univariable analysis. Moreover, univariable analysis primarily aids in identifying which covariates have statistically significant crude estimates (reflecting possible effects on the outcome independent of the main predictor, 2019 GDP per capita), and thus need to be controlled for. Controlling for significant covariates in multivariable analysis, we obtained a positive significant association between 2019 GDP per capita and daily number of RT-PCR tests performed. A previous study [6] investigated the relationships between income distribution metrics, the number of COVID-19 tests, and COVID-19-related mortality on the premise that in regions with high degrees of income inequality, COVID-19 cases were underreported not because of low infection rates but rather because of low testing rates. Consistent with our study results, the authors likewise reported the same positive relationship between GDP per capita and COVID-19 testing rates after controlling for various country-level indicators, albeit a set different from ours (P80/P20 ratio, Gini coefficient, human development index, and extreme poverty rate). It is worth noting that while the SEA region has diverse markets that provide investors with a wide range of economic opportunities, majority of its countries belong to the lower-middle income group [60]. During the period in consideration, many neither had the diagnostic kits readily available nor the technology to produce them, with the supply chain becoming a very important parameter determining how many tests may be conducted [61]. Nations with poorer GDP growth had a harder time procuring testing kits compared to those with better economic profiles, presumably because the latter would tend to have better credit ratings that enable them to obtain loans to finance initiatives and facilities aimed at responding to the health crisis, as discussed earlier. Variability in the number and distribution of testing centers occurring as a function of the number of available kits, as well as accessibility to these testing centers, further impacted testing rates during the earlier part of the pandemic. Moreover, health inequalities occurring within countries may also play a role in testing capacity [62]. Populations that already faced barriers to healthcare access, such as those living in low-income neighborhoods or rural areas, were more likely to face difficulties in accessing COVID-19 testing due to factors such as limited testing facilities in their areas or a lack of transportation to testing centers [44], thereby contributing to unequal health outcomes through hampered case recognition and inadequate treatment [6]. It is important to briefly discuss as well that although a negative association between 2020 GDP per capita and daily number of RT-PCR tests performed persisted in the multivariable analysis, its influence on the outcome of interest is plausibly only partial at most. Nonetheless, its effect still needed to be controlled for in the analysis. Moreover, as GDP per capita is a lagging economic indicator in general, by the time the 2020 figures have been released, changes in testing and resource allocation strategies possibly unrelated to economic performance (such as higher resource allocation towards vaccine development and an increased use of COVID-19 rapid antigen testing) may have already taken place and could constitute reasons behind driving this negative association.

After controlling for covariate effects, we observed a significant inverse relationship between HE per capita and daily number of RT-PCR tests where a more intuitive positive relationship was expected. This can be explained by several factors. First, the rising number of COVID-19 cases necessitating hospital confinement, isolation, and intensive care services had to be identified and established to prevent further spread of disease, ramping up expenditure in the process. Second, there existed scarcity of diagnostic kits and materials such as masks and personal protective equipment (PPE) at the start of the pandemic. This was further exacerbated by export restrictions of COVID-19 tests, treatments and PPE imposed by foreign governments supposedly due to domestic shortages pushing up world prices (e.g., then-United States president Donald Trump imposed a trade policy that sought to redirect surgical masks manufactured by 3M in other countries to the US, and to stop exporting masks manufactured by the US [63]). Additionally, global markets for crucial WHO-designated products to address COVID-19 became highly concentrated during the initial periods of the pandemic [64]. The US, European Union, China, Japan, and South Korea accounted for roughly 80% of total imports. This caused countries to compete based on their respective means, regardless of need. Initially, there was no stable supply chain for diagnostic tests, reagents, and other equipment [61], pushing costs higher, invariably limiting the number of tests that can be conducted. Third, there was a lack of accredited testing laboratory centers and trained personnel to handle laboratory specimens during the earlier months of the pandemic [65]. This prompted governments to initially invest in establishing testing laboratories and in training personnel to conduct these tests over procuring the testing kits themselves. Lastly, the need to identify, isolate, and quarantine suspected cases based on epidemiologic risk, as well as identify places to designate as quarantine facilities, entailed allocating expenses and resources away from testing kit procurement. Traditionally, health authorities recommend that at least 5% of the GDP is spent on healthcare [66]. However, this 5% figure that first appeared in a 1981 WHO document [67] stated that this is an indicator that should be monitored, not as a recommended level of health spending. Nonetheless, government health expenditure grew by around 5% in 2020 in response to these additional needs, effectively increasing the proportion of GDP intended for health spending to 9.7% from 8.8% in the previous year [68]. An important limitation of health expenditure reported as a single metric, however, is that it cannot provide any detail as to what particular aspects of healthcare governments have spent on, or whether there was more public spending on preventive measures to address the pandemic. Additionally, the percentage of GDP utilized for healthcare that a country officially reports underestimates the amount it actually spends on healthcare as this generally reflects just the government’s spending and ignores private health spending. The approach of many countries regarding testing has been contingent on parameters such as incidence, test positivity, hospitalization, and mortality rates [69], and in territories that adopted early border closures, heightened surveillance of arriving passengers at airports and other points of entry, and prompt isolation and treatment of suspected cases, transmission was thwarted and case numbers remained low, necessitating lesser testing. Thailand, celebrated as one of the earlier COVID-19 success stories in SEA, invested in targeted surveillance strategies during the pandemic’s second wave, allowing them to concentrate testing to high-risk venues and vulnerable populations (i.e., healthcare workers, public transportation operators, delivery workers, migrant workers, and informal settlers) [70]. In addition to swift flight suspension, early border control, mass surveillance and strict NPI implementation, Vietnam, highly praised for its innovative and effective management of the first pandemic wave, managed to develop its own quality and affordable (i.e., less than US$25) COVID-19 test kits despite limited resources [71]. Vietnam’s robust response is likewise credited to its wealth of experience dealing with the SARS epidemics of 2003 and human cases of avian influenza between 2004 and 2010 [72]. Putting stringent preventive measures in place during the first months of the pandemic, when RT-PCR testing was still in short supply, may have likewise accounted for this observed inverse relationship between HE per capita and testing rates because testing kits had to be used sparingly. As economies recovered, and as testing strategies and COVID-19 case definitions changed over time with the increasing availability of acceptable alternatives to RT-PCR such as rapid antigen testing, this relationship may have reversed accordingly.

Earlier, we argued that any association between CPI and daily RT-PCR testing observed on crude analysis could be partly driven by possible independent effects of accountability, transparency, public trust, stringency of pandemic measures, and degree of decentralization, thus the need to control for such factors. Naturally, relevant country-level metrics corresponding to these factors become necessary if we are to tease out a more definitive quantifiable relationship between CPI and RT-PCR testing rates. Unfortunately, not all these metrics are available to aid in the analysis, as mentioned earlier. While the CPI incorporates in its measure certain aspects of accountability, transparency, and degree of public trust [27, 28], it is still based on subjective perceptions (which may be readily influenced by mass media, cultural biases, and personal experiences) rather than more concrete evidence of corruption. Pratolo et al [37] quantified accountability, transparency, and degree of public trust utilizing a Likert-scale questionnaire adapted and validated for the COVID-19 pandemic situation, but these measurements were done at the village government level rather than at the national level. In our study, we observed significant positive relationships of SI and RAI with testing rates. During the early months of the pandemic, mandating frequent RT-PCR testing as part of stringency measures (e.g., with incoming travelers and returning citizens from overseas for case detection and quarantine purposes, as well as with essential workers to avoid disruption of essential services during the pandemic) may have resulted in higher testing rates in some countries. A concurrent increase in local officials’ accountability presumably compelled by public scrutiny and the urgency of case-finding to curb viral spread may have likewise facilitated timely distribution of RT-PCR tests and other health services to subnational units in more decentralized governments, though this may be complicated by the undesired tendency of services being overprovided to socioeconomic strata that can afford them [49]. Even after accounting for the effects of SI and RAI, as well as the other covariates in the full regression model, a negative association remains between CPI and daily RT-PCR testing. One possible explanation for this is that some countries with lower CPIs (i.e., higher perceived corruption) may have received greater international assistance or funding specifically aimed at strengthening their healthcare systems and COVID-19 response. Additionally, while corrupt practices are expected to divert resources away from public health efforts, some governments with high levels of perceived corruption may have been able to allocate more resources to testing as a means of bolstering their legitimacy, to show they are taking decisive action to protect their citizens’ well-being despite the perceived corruption [73]. By focusing on healthcare through leveraging the distribution of resources, corrupt governments can gain public support and potentially mitigate dissatisfaction resulting from corruption allegations. Capitalizing on tangible outputs can likewise serve as diversionary tactics, shifting public attention away from corruption allegations [73]. Corrupt governments may have likewise faced increased scrutiny from the media, civil society organizations, and public watchdogs, resulting in greater attention and pressure to respond effectively to the pandemic. By emphasizing their response to the pandemic, governments may try to divert scrutiny and criticism, presenting themselves as competent and responsible authorities, even if corruption persists in other areas.

Our study has several limitations. As discussed earlier, GDP per capita does not directly reflect the inequalities in a country’s income distribution or the costs imposed on human health [7], and that such income inequalities, brought to the surface by the COVID-19 pandemic, may also play a role in differences in testing rates across countries. The Gini coefficient is often used alongside GDP per capita to quantify the degree of inequality in income distribution, with its possible values ranging from 0 (indicating perfect equality) to 1 (indicating maximal inequality). While there are no studies that correlate the Gini coefficient with testing capacity or intensity, it has significant associations with COVID-19 disease burden and deaths, even after controlling for testing intensity [74, 75]. Researchers pointed out enhancements of RT-PCR testing as one of the interventions that can be targeted and improved on in areas with high income inequality to control viral spread [74], suggesting that in such areas, easier access to RT-PCR testing might be confined mostly to sections of the population that can afford them. While including the Gini coefficient as a covariate in our analysis would have been more informative, many countries do not have up-to-date information (and some not having any information at all) on this metric. Therein lies another important limitation of our study. Brunei, for example, “does not maintain a regular schedule of economic data releases, and is notoriously late with any data releases,” as stated in a 2011 report [8]. To date, no information on the Gini coefficient has been released by Brunei. Additionally, it has been the experience of densely populated countries with relatively larger land masses and relatively lower GDPs per capita to ration their testing kits, as there are not enough to go around, necessitating the need to prioritize symptomatic patients [59, 76]. Besides not being able to include the Gini coefficient as a covariate for reasons discussed, another limitation involves the inclusion of only 11 countries in the regression model, which resulted in some issues of covariate representativeness. For example, higher values of GDP per capita and CPI had lesser country representation in our data than lower values. However, given that the structure of our daily RT-PCR testing data is panel instead of cross-sectional, it enjoys the advantages of containing more degrees of freedom and more sample variability that improves the efficiency of coefficient estimation [77], thus possibly offsetting the limitation of having a small number of countries included in the study. Also, as with any regression analysis, ecological regression carries the potential for spurious statistical estimates due to unmeasured confounding [78]. Panel data, however, minimizes the impact of omitted-variable bias because it contains information on the intertemporal dynamics and the individuality of the daily testing counts that allows for control of the effects of missing or unmeasured confounders [79]. Additionally, the fixed-effects approach allows for isolating the time-varying effects of independent variables on the outcome while holding unobserved time-invariant factors constant, thereby minimizing omitted-variable bias as well. Yet another limitation of our study is the lack of provisions in the analysis to account for COVID-19 pooled testing. Pooled testing is a useful time- and cost-efficient strategy to continuously monitor large populations during the pandemic [80], particularly in areas where the number of positive tests is expected to be low, and is a worthwhile approach to incorporate in the analysis. However, available data on daily RT-PCR testing were on tests done on an individual basis. Liu [81] describes an optimization technique commonly employed in economics and management wherein: a theoretical model linking the local population tested for COVID-19 in a specific region, the quantity of biological samples that can be incorporated into a single test, along with the associated resource cost and time functions is constructed; numerical simulation outcomes are leveraged to establish both the resource cost and time functions and; a minimizable loss function is devised, and the optimal quantity of included samples is computed. Unfortunately, due to the ecological design of our study, it may be problematic to incorporate such optimizing techniques as local instead of country-level COVID-19 infection probabilities need to be introduced. Additionally, while the dynamic nature of such infection probabilities can be handled by panel data regression, such data during the first 6 months of the pandemic are not readily available to the best of our knowledge. While positivity rates can serve as surrogate for infection probabilities, the true number of positive cases may most likely be underestimated [4], as discussed previously. Finally, another limitation is in reference to our study’s ecological design, wherein measures of exposure are only a proxy based on population means. As such, inferences about the nature of individuals should not be deduced using population-level data.

## Conclusion and recommendations

In our study, we found a statistically significant direct relationship between 2019 GDP per capita and daily RT-PCR testing on multivariable analysis. We likewise identified country-level indicators significantly associated with differences in RT-CPR testing rates across SEA countries. Overall, modeling of RT-PCR testing rates observed during the beginning of the pandemic as a function of 2019 GDP per capita and these various country-level indicators highlights the need for increased investment in healthcare infrastructure and resources, as well as targeted efforts to address health inequalities and improve access to healthcare for all populations. Due to this study’s ecological design, we caution on applying our results to the individual level. We likewise urge the readers to regard this study as exploratory and hypothesis-generating at best, with the hope that the observed statistical relationships would lead to further investigation of the involved country-level indicators at the individual level using methodologies beyond conventional regression analysis (including optimization of COVID-19 pooled testing for surveillance) in order to guide policy makers regarding what needs to be improved on for pandemic preparedness.

## Supporting information

S1 Checklist STROBE checklist. (DOCX)
