# Supplementary material for: Determinants of differences in RT-PCR testing rates among Southeast Asian countries during the first six months of the COVID-19 pandemic
Source: PLOS Glob Public Health. 2023 Nov 7;3(11):e0002593. doi: 10.1371/journal.pgph.0002593 (PMC10629619; doi:10.1371/journal.pgph.0002593)
Supplement: S1 Checklist — (DOCX) [file pgph.0002593.s001.docx]

STROBE Statement—checklist of items that should be included in reports of observational studies

|  | Item No. | Recommendation | Page  No. | Relevant text from manuscript |
| --- | --- | --- | --- | --- |
| **Title and abstract** | 1 | (*a*) Indicate the study’s design with a commonly used term in the title or the abstract | 2 | ‘Longitudinal ecological study’ as stated in the *Abstract*. |
|  |  | (*b*) Provide in the abstract an informative and balanced summary of what was done and what was found | 2 | ‘Using open-access COVID-19 panel data, we estimated the effect of various indicators (…) on daily COVID-19 testing by performing random-effects negative binomial regression.’  ‘… A rise in GDP per capita by 100 international dollars is associated with a 15.66% increase in the number of daily tests performed.’ |
| Introduction | | | |  |
| Background/rationale | 2 | Explain the scientific background and rationale for the investigation being reported | 3–4 | ‘Pre-pandemic, the SEA region’s rising economy (driven by a combination of domestic consumption, foreign investment, and exports) saw an optimistic outlook notwithstanding a then-forecasted slight drop in real gross domestic product (GDP) growth to 6.1% from 6.3% in 2019. Despite these encouraging figures, there emerged a wide variation in COVID-19 testing capacity across SEA countries,’ as stated in the *Introduction*. |
| Objectives | 3 | State specific objectives, including any prespecified hypotheses | 5 | ‘… we aimed to determine if, besides GDP per capita, country-level indicators relating to governance, enforcement of public health measures, and government investment in health and research significantly influenced the observed differences in COVID-19 testing rates among SEA countries during the first six months of the pandemic’ as stated in *Introduction*. |
| Methods | | | |  |
| Study design | 4 | Present key elements of study design early in the paper | 5 | ‘This is a longitudinal ecological study that made use of open-access data obtained from Our World in Data (OWID) (…), World Bank Open Data (…),Transparency International (…), and the Cadmus European Institute Research Repository (…)’ as stated in *Methods* under subheading *Study design, setting, and data sources*. |
| Setting | 5 | Describe the setting, locations, and relevant dates, including periods of recruitment, exposure, follow-up, and data collection | 5­–6 | ‘In accordance with our problem statement and study objective, we limited our scope to data obtained from the eleven SEA countries (…), and limited the period of our analysis to the first six months of the pandemic, starting on and including 13 January 2020. We concentrated on the first six months of the COVID-19 pandemic because it was during this period when governments of different nations were at the height of mobilizing resources to implement planned public health measures in coordination with the different sectors in the race to control virus spread, whilst periodically evaluating and modifying these implementations to come up with more effective plans of action’ as stated in *Methods* under subheading *Study design, setting, and data sources*. |
| Participants | 6 | (*a*) *Cohort study*—Give the eligibility criteria, and the sources and methods of selection of participants. Describe methods of follow-up  *Case-control study*—Give the eligibility criteria, and the sources and methods of case ascertainment and control selection. Give the rationale for the choice of cases and controls  *Cross-sectional study*—Give the eligibility criteria, and the sources and methods of selection of participants | N/A | [In our ecological study, the unit of observation is country instead of individual study participants. Limiting our scope to the SEA region, 11 countries are included in the study.] |
|  |  | (*b*) *Cohort study*—For matched studies, give matching criteria and number of exposed and unexposed  *Case-control study*—For matched studies, give matching criteria and the number of controls per case | N/A |  |
| Variables | 7 | Clearly define all outcomes, exposures, predictors, potential confounders, and effect modifiers. Give diagnostic criteria, if applicable | 8–22 | Study variables defined in the *Methods* under subheadings *Dependent variable* (page 8), *Explanatory variables* (pages 8–20), and *Other variables* (pages 20–22). |
| Data sources/ measurement | 8* | For each variable of interest, give sources of data and details of methods of assessment (measurement). Describe comparability of assessment methods if there is more than one group | 5 | ‘… open-access data obtained from Our World in Data (OWID) (…), World Bank Open Data (…), Transparency International (…) and the Cadmus European Institute Research Repository (…)’ as stated in *Methods* under subheading *Study design, setting, and data sources* (page 5). |
| Bias | 9 | Describe any efforts to address potential sources of bias | 38–41 | Addressed in the last paragraph in *Discussion* (limitations; pages 38–41). |
| Study size | 10 | Explain how the study size was arrived at | 5 | [In our ecological study, the unit of observation is country instead of individual study participants.]  ‘…we limited our scope to data obtained from the eleven SEA countries (Brunei, Cambodia, Indonesia, Laos, Malaysia, Myanmar, Philippines, Singapore, Thailand, Timor-Leste, and Vietnam)’ as stated in *Methods* under subheading *Study design, setting, and data sources*. |
| Quantitative variables | 11 | Explain how quantitative variables were handled in the analyses. If applicable, describe which groupings were chosen and why | 22–23 | Addressed in *Methods* under subheading *Data gathering, preparation and cleaning*. |
| Statistical methods | 12 | (*a*) Describe all statistical methods, including those used to control for confounding | 23–26 | Addressed in *Statistical analysis*. |
|  |  | (*b*) Describe any methods used to examine subgroups and interactions | N/A |  |
|  |  | (*c*) Explain how missing data were addressed | 23 | ‘Missing data on country-level indicators not requiring multiple metrics or complex methods for derivation (…), if any, were imputed by regressing the indicator in question on the other country-level indicators, including population density. Robust regression (*…*) was used for this purpose in anticipation of extreme indicator values, as it is less sensitive to outliers compared to standard linear regression’ as stated in *Methods* under subsection *Study design, setting, and data sources*. |
|  |  | (*d*) *Cohort study*—If applicable, explain how loss to follow-up was addressed  *Case-control study*—If applicable, explain how matching of cases and controls was addressed  *Cross-sectional study*—If applicable, describe analytical methods taking account of sampling strategy | N/A |  |
|  |  | (*e*) Describe any sensitivity analyses | N/A |  |
| Results | | | | |
| Participants | 13* | (a) Report numbers of individuals at each stage of study—eg numbers potentially eligible, examined for eligibility, confirmed eligible, included in the study, completing follow-up, and analysed | N/A | [None of the included 11 countries were excluded at any point during the research period of interest.] |
|  |  | (b) Give reasons for non-participation at each stage | N/A |  |
|  |  | (c) Consider use of a flow diagram | N/A |  |
| Descriptive data | 14* | (a) Give characteristics of study participants (eg demographic, clinical, social) and information on exposures and potential confounders | 26–29 | In our ecological study, the unit of observation is country instead of individual study participants. The country-specific variables and indicators were described in the first paragraph under *Results* (page 26) and summarized in Table 1 (pages 27–28; table legend on pages 28–29). |
|  |  | (b) Indicate number of participants with missing data for each variable of interest | 27 | ‘Of note, Timor-Leste did not have information on R&D expenditure and number of researchers per one million population, and were thus imputed by regressing each missing indicator on the other country-level indicators, including population density, as described previously’ as stated in *Results*. |
|  |  | (c) *Cohort study*—Summarise follow-up time (eg, average and total amount) | N/A |  |
| Outcome data | 15* | *Cohort study*—Report numbers of outcome events or summary measures over time | 26–29 | The total number of RT-PCR tests performed are described in the first paragraph of *Results* (page 26) and summarized in Table 1 (pages 27–28; table legend on pages 28–29). |
|  |  | *Case-control study—*Report numbers in each exposure category, or summary measures of exposure | N/A |  |
|  |  | *Cross-sectional study—*Report numbers of outcome events or summary measures | N/A |  |
| Main results | 16 | (*a*) Give unadjusted estimates and, if applicable, confounder-adjusted estimates and their precision (eg, 95% confidence interval). Make clear which confounders were adjusted for and why they were included | 29–31 | Addressed in the second paragraph of *Results* (pages 29–30) and Table 2 (pages 30–31). |
|  |  | (*b*) Report category boundaries when continuous variables were categorized | N/A |  |
|  |  | (*c*) If relevant, consider translating estimates of relative risk into absolute risk for a meaningful time period | N/A |  |
| Other analyses | 17 | Report other analyses done—eg analyses of subgroups and interactions, and sensitivity analyses | N/A |  |
| Discussion | | | | |
| Key results | 18 | Summarise key results with reference to study objectives | 31–38 | Key study results were summarized at the beginning of each of the first three paragraphs of *Discussion*. |
| Limitations | 19 | Discuss limitations of the study, taking into account sources of potential bias or imprecision. Discuss both direction and magnitude of any potential bias | 38–41 | Addressed in the final paragraph of *Discussion*. |
| Interpretation | 20 | Give a cautious overall interpretation of results considering objectives, limitations, multiplicity of analyses, results from similar studies, and other relevant evidence | 31–38 | Key study results were discussed and interpreted in light of available literature in the first three paragraphs of *Discussion*. |
| Generalisability | 21 | Discuss the generalisability (external validity) of the study results | 31–38 | Addressed in the first three paragraphs of *Discussion*. |
| Other information | |  | | |
| Funding | 22 | Give the source of funding and the role of the funders for the present study and, if applicable, for the original study on which the present article is based | N/A | No funding |

*Give information separately for cases and controls in case-control studies and, if applicable, for exposed and unexposed groups in cohort and cross-sectional studies.

**Note:** An Explanation and Elaboration article discusses each checklist item and gives methodological background and published examples of transparent reporting. The STROBE checklist is best used in conjunction with this article (freely available on the Web sites of PLoS Medicine at http://www.plosmedicine.org/, Annals of Internal Medicine at http://www.annals.org/, and Epidemiology at http://www.epidem.com/). Information on the STROBE Initiative is available at www.strobe-statement.org.
